# Testicular Pain and Mesenteric Adenitis as an Atypical Presentation of COVID-19

**DOI:** 10.7759/cureus.15956

**Published:** 2021-06-27

**Authors:** Shreya Desai, Dennis Citrin, Mark Conneely

**Affiliations:** 1 Internal Medicine, Rosalind Franklin University of Medicine and Science, North Chicago, USA; 2 Hematology and Oncology, Captain James A. Lovell Federal Health Care Center, North Chicago, USA; 3 Radiology and Nuclear Medicine, Captain James A. Lovell Federal Health Care Center, North Chicago, USA

**Keywords:** covid-19, sars-cov-2 (severe acute respiratory syndrome coronavirus -2), mesenteric adenitis, asymptomatic covid-19, testicular pain

## Abstract

A 21-year-old Caucasian male with no past medical history presented to the emergency department with right lower quadrant pain radiating to the right testicle for two days. He reported an occasional dry cough that day but denied any fever or other infectious symptoms. The patient was afebrile with a normal physical examination. CT of the abdomen and pelvis showed prominent right lower quadrant lymphadenopathy. Viral panel for common respiratory pathogens returned negative. A nasopharyngeal swab for SARS-CoV-2 by Xpert® Xpress SARS-CoV-2 reverse transcriptase-polymerase chain reaction (Cepheid Inc., Sunnyvale, CA) was positive. The patient remained in quarantine for 14 days. He was reevaluated seven weeks later with spontaneous resolution of his abdominal pain and the continued absence of upper respiratory symptoms. A repeat CT scan seven weeks later showed persistent mesenteric lymphadenopathy. Repeat COVID-19 testing was not performed at this time. While the frequency of atypical presentation of COVID-19 remains unknown, healthcare providers must continue to remain vigilant and consider COVID-19 as a differential diagnosis in any patient presenting to the emergency department despite the lack of respiratory and gastrointestinal symptoms. Further research is warranted to examine the possibility of asymptomatic spread in asymptomatic patients with persistent radiologic findings and to assess whether repeat COVID-19 testing is warranted in such patients.

## Introduction

The COVID-19 pandemic has caused a global health crisis that continues to ravage nations across the world. In the time of scarce medical supplies and personal protection equipment (PPE) shortage, asymptomatic or atypical presentations of COVID-19 can lead to unintentional exposure by the healthcare workers (HCW). There continues to be growing evidence regarding atypical presentations of COVID-19 infection. Here we present a case report of a man who was referred to the emergency department for testicular pain and tested positive for COVID-19 infection. He was subsequently found to have mesenteric lymphadenopathy which persisted for weeks after the resolution of the infection. 

## Case presentation

**Figure 1:**
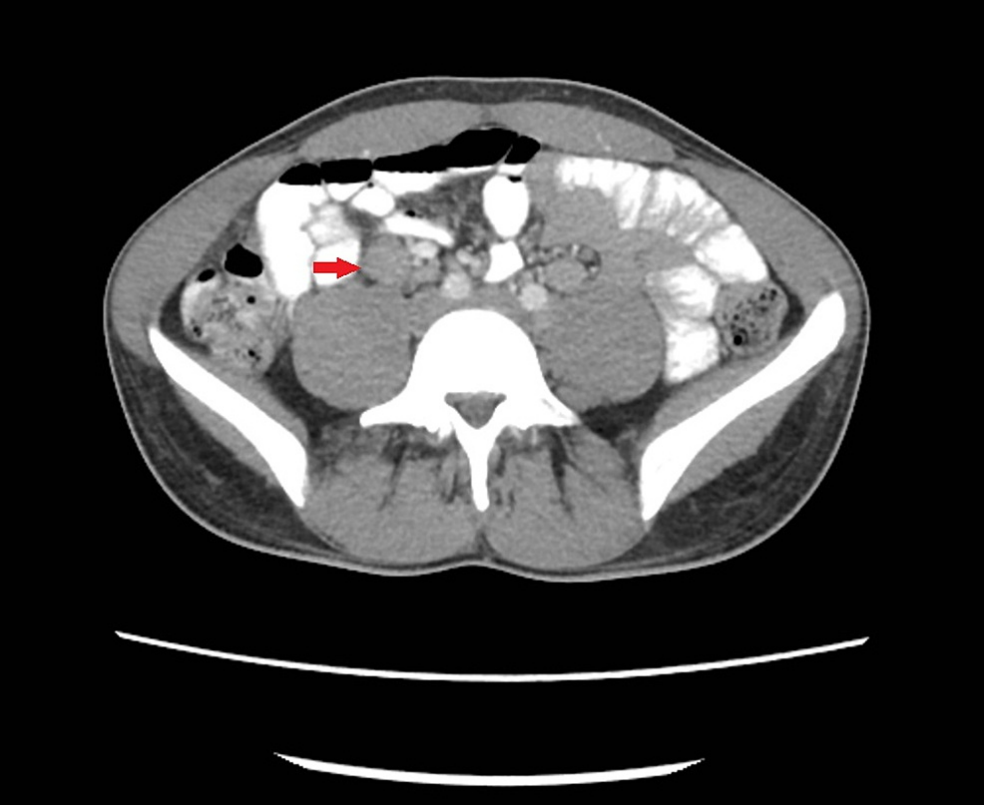
Axial image from CT of the abdomen and pelvis with oral and IV contrast demonstrates a cluster of enlarged mesenteric lymph nodes in the right lower quadrant (red arrow).

**Figure 2:**
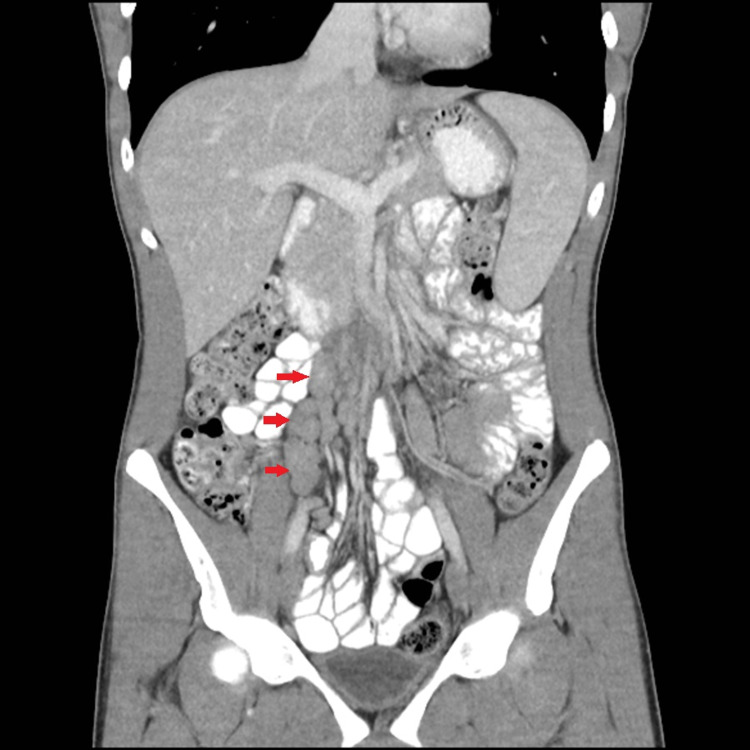
Coronal reformatted image from CT of the abdomen and pelvis with oral and IV contrast demonstrates a cluster of enlarged mesenteric lymph nodes in the right lower quadrant (red arrows).

A 21-year-old Caucasian male with no past medical history presented to the emergency department with right lower quadrant pain radiating to the right testicle for the past two days. He reported occasional dry cough that day but denied any fever, sore throat, rhinorrhea, body aches, vomiting, diarrhea, loss of taste or smell. He had no history of testicular torsion or abdominal trauma. The patient denied recent sick contacts. He was afebrile with normal vital signs. Examination of the lymph nodes, abdomen, and testes/scrotum was normal. Laboratory findings were unremarkable and did not show elevated inflammatory markers or lymphopenia. The computed tomography (CT) of the abdomen and pelvis showed prominent right lower quadrant lymphadenopathy (Figure 1, 2). 

**Figure 3:**
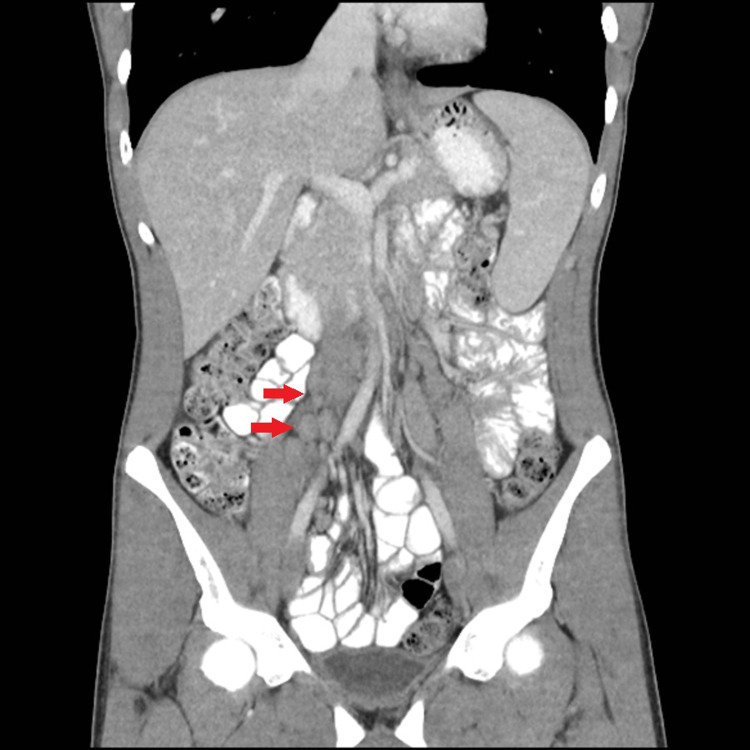
Coronal reconstructed image from CT abdomen and pelvis performed seven weeks after the initial CT shows stable enlarged mesenteric lymph nodes in the right lower quadrant (red arrows).

The workup for bacterial etiology was negative. Viral panel for common respiratory pathogens including influenza A, influenza B, adenovirus, rhinovirus, respiratory syncytial virus, and human metapneumovirus returned negative. A nasopharyngeal swab for SARS-CoV-2 by Xpert® Xpress SARS-CoV-2 reverse transcriptase-polymerase chain reaction (Cepheid Inc., Sunnyvale, CA) was positive. Due to the lack of overt flu-like symptoms or respiratory distress, the patient did not receive any medical therapy for SARS-CoV-2. The patient remained in quarantine for 14 days and was subsequently released from isolation. He was reevaluated seven weeks later at which point his abdominal pain was resolved. He remained free of fever, respiratory, or gastrointestinal symptoms. Physical examination of the abdomen and testes/scrotum was normal. A repeat CT scan seven weeks later showed persistent mesenteric lymphadenopathy (Figure 3). 

Repeat COVID-19 testing was not performed at this time due to limited testing availability and due to the patient's lack of symptoms. CT scan performed four months after initial diagnosis showed resolution of the lymphadenopathy. 

## Discussion

Acute mesenteric lymphadenitis is a self-limiting inflammatory condition typically occurring in children, adolescents, and young adults [1]. On imaging, it often presents as right-sided lymphadenopathy and may often be associated with the underlying inflammatory or infectious disease process. Reports of mesenteric adenitis have been previously reported in the pediatric population with severe COVID-19 infection who developed multisystem inflammatory syndrome in children (MIS-C), a condition characterized by systemic hyper-inflammation with multiorgan damage. A retrospective analysis of 44 pediatric patients showed that 84.1% of patients with MIS-C presented with gastrointestinal (GI) symptoms with 100% accompanied by fever and 25% of patients required supplemental oxygen [2]. Two patients were noted to have mesenteric lymphadenopathy [2]. In contrast, mesenteric adenitis has not been frequently reported among adults, especially among asymptomatic patients. Remarkably, our patient had no respiratory or gastrointestinal symptoms apart from mild abdominal and testicular pain. Further workup regarding painless lymphadenopathy was not performed as his lymphadenopathy eventually resolved in the months after his COVID-19 diagnosis. Testicular pain and painless mesenteric lymphadenopathy have been rarely reported as presenting symptoms of COVID-19 infection in the literature and may be due to persistent immune stimulation [3,4,5]. In such patients, the significance of prolonged lymphadenopathy raises the possibility of persistent infection with concern for asymptomatic spread. Repeat testing should be ordered in such patients for continued observation. Additionally, the long-term implications of presumed immune stimulation causing lymph node enlargement are unknown and warrant long-term observation in such patients. 

## Conclusions

While the frequency of atypical presentation of COVID-19 remains unknown, healthcare providers must continue to remain vigilant and consider COVID-19 as a differential diagnosis in any patient presenting to the emergency department despite the lack of respiratory and gastrointestinal symptoms. Considerations ought to be given to testing of such atypical or asymptomatic patients to avoid further community spread. Long-term implications of presumed immune stimulation causing lymph node enlargement are unknown and require long-term observation.
